# Tuning Lanthanide Binding with Phenanthroline-Based Diamides via Electron-Donating and Electron-Withdrawing Groups

**DOI:** 10.1021/acs.jpca.5c03506

**Published:** 2025-10-20

**Authors:** Anton S. Pozdeev, Alexander S. Ivanov, Santa Jansone-Popova, De-en Jiang

**Affiliations:** † Department of Chemical and Biomolecular Engineering, 5718 Vanderbilt University, Nashville, Tennessee 37235, United States; ‡ Chemical Sciences Division, 6146 Oak Ridge National Laboratory, Oak Ridge, Tennessee 37831, United States

## Abstract

Efficient separation of lanthanides is challenging due to their similar sizes and properties but continues to attract intensive theoretical and experimental interest. 1,10-phenanthroline-2,9-diamide (DAPhen) ligands are promising in solvent extraction separations of f-elements due to their combinations of soft N and hard O donors, but how the conjugated electrons and charge densities on the N and O donors can be further manipulated to tune the binding with lanthanides has not been fully explored. By substituting hydrogen atoms on the phenanthroline (Phen) skeleton with various electron-donating and electron-withdrawing groups, here we assess their impact on the complexation properties crucial for lanthanide separation. Employing advanced quantum chemical techniques, we have found that these substitutions significantly affect binding energies, although they have a relatively weak influence on selectivity for the whole lanthanide series. By elucidating the bonding nature in the studied lanthanide complexes, we provide a unified interpretation of these effects, aiming to comprehend the potential role of different electron-donating and electron-withdrawing substituents in lanthanide extraction and separation. These insights provide valuable guidelines for the rational design of DAPhen-based ligands for improved rare-earth element separations.

## Graphical abstract

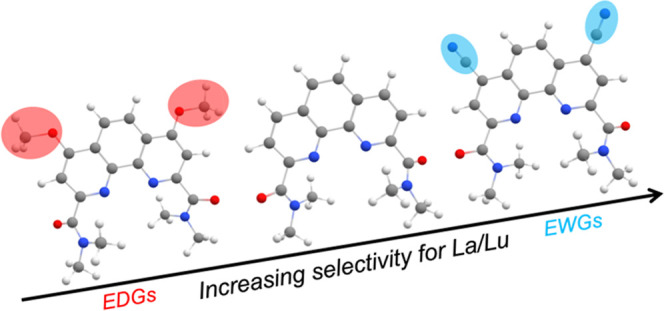

## 1 Introduction

Rare-earth elements (REEs), which include yttrium (Y), scandium (Sc), and the 15 lanthanides (Ln), are essential for numerous modern industries^1,2^ and medical applications.^3,4^ For example, neodymium (Nd) is recognized as a critical material^5^ and a key component in permanent magnets.^6^ Certain synthetic, short-lived radioactive isotopes like Tb-161 and Lu-177 play essential roles in cancer treatment in radiopharmacology.^7^ Gadolinium (Gd) is widely used as a contrast agent in magnetic resonance imaging.^8^ Given their extensive and strategic applications, the efficient separation of REEs remains a critical challenge, classified as one of the seven chemical separations to change the world.^9^ Productive separation processes are not only a technical issue but also strategic imperatives to ensure a stable and secure supply of these essential elements.^10^

Solvent extraction remains the predominant industrial method for REE separation;^11,12^ however, due to the similar chemical and physical properties of their trivalent ions, efficient separation relies on carefully exploiting subtle differences in ionic radii.^13^ Recently, density functional theory (DFT) computations have been applied to study selective complexation of lanthanides and actinides by electronically tuned ligands and to explore the influences of subtle electronic modifications of ligand frameworks on the covalency, orbital interactions, and thus the complexation and separation selectivity toward lanthanides vs actinides.^14,15^ This approach has also been used to evaluate ligand electronic tuning in enhancing the selectivity and efficiency of REE separation processes.^12,16^

Ligands that combine hard O-donor and soft N-donor atoms, such as derivatives of 1,10-phenanthroline-2,9-dicarboxylic acid (PDA) including 1,10-phenanthroline-2,9-diamide (DAPhen; Figure 1), have been found highly effective for the extraction and separation of lanthanides.^17–19^ These ligands provide efficient and rapid complexation of lanthanides, forming complexes soluble in organic solvents.^20^ Modifications of the DAPhen ligands include exchange of the substituents on the amide groups.^16,21,22^ An alternative but less popular approach involves installing various electron-withdrawing groups (EWGs) such as –F,^23^ –Cl,^24^ –CF_3_,^25^ and –NO_2_,^26^ as well as electron-donating groups (EDGs) like alkyl substituents^27^ and alkoxy groups^28^ in the phenanthroline backbone.^14,15,29,30^ All these modifications change the electron distribution and density at the four N/O donors of DAPhen and, therefore, influence different extraction properties, including the effectiveness of separating light vs heavy lanthanides,^31^ lanthanides vs actinides,^32–34^ and solubility in water or organic phase.^35,36^ As a result, they bring new opportunities for innovations in REE separations.^37^

**Figure 1:**
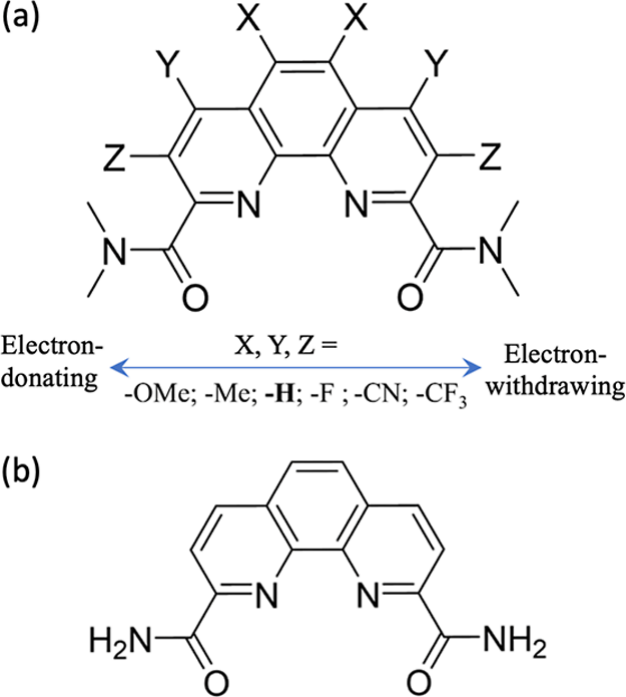
(a) Schematic representation of symmetric *X*, *Y*, and *Z* positions in the 1,10-phenanthroline-2,9-diamide (DAPhen) backbone (*N*,*N*,*N*′,*N*′-tetramethyl-1,10-phenanthroline-2,9-dicarboxamide, TMeDAPhen) and the list of chosen EDGs and EWGs; (b) the reference DAPhen ligand.

Despite previous studies examining modifications of phenanthroline-based ligands for Ln separations, systematic computational investigations specifically addressing how EDGs and EWGs on the phenanthroline backbone influence lanthanide binding energies and selectivity remain limited. Consequently, a detailed understanding of how electronic tuning at specific backbone positions affects Ln­(III) complexation and extraction behavior is lacking. The current work addresses this research gap by systematically substituting hydrogen atoms in the phenanthroline backbone of DAPhen ligands with various EDGs and EWGs (Figure 1) and employing advanced computational chemistry tools to investigate the selectivity trends. Furthermore, to elucidate electronic structure effects in detail, we utilize the quantum theory of atoms in molecules (QTAIM),^38^ a sophisticated bonding analysis widely used in computational chemistry.^39–41^ By clarifying these electronic effects, our study provides a unified explanation of the role of EDGs/EWGs in lanthanide complexation.

## 2 Computational Methods

All the Kohn–Sham DFT calculations were performed using ORCA 5.04 software package^42^ with advanced “defgrid3” settings for integration grids and C_1_ point group symmetry. For SCF convergence, we used the TightSCF convergence threshold for optimization and the VeryTightSCF threshold for single-point (SP) and frequency calculations. For optimization, we used the TightOpt convergence threshold. The RI-J approximation was utilized to accelerate energy and force calculations. Additionally, to address the limitations of the harmonic oscillator approximation at low frequencies, entropic contributions to free energies were calculated using the Grimme’s Quasi-RRHO approach.^43^

The complex structures were optimized by applying the restricted DFT formalism with the PBE (Perdew–Burke–Ernzerhof)^44^ functional of GGA quality and the def2-SVPD basis set,^45^ in conjunction with the 4f-in-core Stuttgart/Dresden (SDD) pseudopotential^46,47^ and corresponding MWB-I basis set on Ln atoms.^48^ We note that the present use of 4f-in-core pseudopotentials does have some limitations, including neglect of magnetism and spin–orbit coupling^49^ as well as lack of subtle description of covalency; nevertheless, it offers a good balance of computational efficiency and accuracy to examine the overall ligand-complex trends for the many complexes (over 200) in the present work. Grimme’s D3 dispersion correction with Becke-Johnson damping (D3BJ)^50^ and the effects of implicit solvents via the conductor-like polarizable continuum model (CPCM)^51^ were included. All optimized geometries were checked via nuclear Hessian calculations, confirming the absence of imaginary frequencies. In the second step, we performed SP energy calculations on the optimized geometries with larger basis sets to improve the energy. We used the more extended MWB-II basis set^52^ for Ln and the def2-TZVPD basis set^45,53^ for other elements. The calculations were performed using the unrestricted DFT formalism in conjunction with the hybrid GGA PBE0 functional,^54^ D3BJ dispersion correction, and SMD solvation model.^55^ Thus, the electronic component of Gibbs energy, unless otherwise stated, was improved by the PBE0 level of theory.

We have investigated the influence of EDGs/EWGs based on two energetic values: binding energy (Δ*G*_b_) according to eq 1 and selectivity (ΔΔ*G*) according to eq 2.^31,56^ In both equations, L_target_ refers to the substituted ligands while L_ref_ refers to the unsubstituted DAPhen ligand with two C­(=O)–NH_2_ groups (Figure 1b). A negative ΔΔ*G* indicates a preference for La­(III) to complex with the target ligand and Ln­(III) to complex with the reference ligand. The more negative the ΔΔ*G* value, the higher the selectivity between La and Ln. In all calculations, we applied the approach of modeling aqueous-phase selectivity across the lanthanide series with an explicit consideration of the first coordination sphere around a Ln cation. The denticities of all nitrates are equal to two. As previously employed by others^57,58^ and by us,^56,59^ the 1:1 DAPhen-to-metal ratio for the Ln­(III) complexes was modeled because it has been found that they are the dominant species during REE separations in high-concentration HNO_3_ solutions (∼3 M) by multidentate ligands such as Phen and DAPhen derivatives.^28,60^ Of note, 2:1 and 3:1 complexes could also form under different conditions.^61,62^ The effect of the outer hydration shells is computed using the CPCM model for optimization and solving the nuclear Hessian task. The SMD model is applied for more accurate SP energy calculations at a more sophisticated level of theory. The initial geometries of the complexes were built manually in molecular modeling software.

1 $$L_{target\left(aq\right)}+Ln({NO}_{3}{)}_{3}(H_{2}{O)}_{3\left(aq\right)}⇄Ln{\left(L_{target}{)(NO}_{3}\right)}_{3\left(aq\right)}+{3H}_{2}O_{\left(aq\right)}$$

2 $${{\left(L_{target}{)(NO}_{3}\right)}_{3\left(aq\right)}⇌La(L_{target}{)(NO}_{3}{)}_{3\left(aq\right)}+Ln\left(L_{ref}{)(NO}_{3}\right)}_{3\left(aq\right)}$$

For the QTAIM analysis, SP calculations were conducted using the second-order Douglas–Kroll-Hess (DKH2) Hamiltonian^63^ with a finite-size Gaussian-like nucleus model.^64^ SARC-DKH-TZVPP (segmented all-electron relativistically contracted) basis set^65^ was employed for La and Lu, while the ma-DKH-def2-TZVP basis set in conjunction with SARC/J^65^ and the AutoAux auxiliary basis sets were applied for all other atoms. As in the second step, we applied the PBE0 functional with D3BJ dispersion correction and accounting for the solvation effects at the SMD level. For simplicity, we denote this scheme as the DKH2-PBE0-D3 level of theory. QTAIM analysis and calculations of partial atomic charges (according to Truhlar’s charge model 5)^66^ were performed using Multiwfn software,^67^ using the.wfn files generated by ORCA as the input. All optimized geometries, harmonic-frequency data, and complete ORCA input for every Ln complex studied were deposited and are freely available in the ioChem-BD Computational Chemistry repository.^68^

## 3 Results and Discussion

### 3.1 Substitution at *X* or *Y* Positions and Effects on Charges

Since the Ln-TMeDAPhen coordination is primarily ionic via the N and O donors, the binding energies are expected to be very sensitive to the partial atomic charges on those donors. Therefore, we first examined how substituents at positions *X* and *Y* affect the partial atomic charges on the N and O donors. As shown in Figure 2, the EDGs make both donors more negatively charged, while the EWGs make them less so, which is expected. Comparing the *X* and *Y* positions, one can see that the N donors, being para to the *Y* positions, are most sensitive to the *Y*-position substitution. Consequently, we expect that EDGs should strengthen the binding between Lns and substituted TMeDAPhen ligands, while EWGs weaken it, and that such changes will be more pronounced for substitution at *Y* than that at *X*.

**Figure 2:**
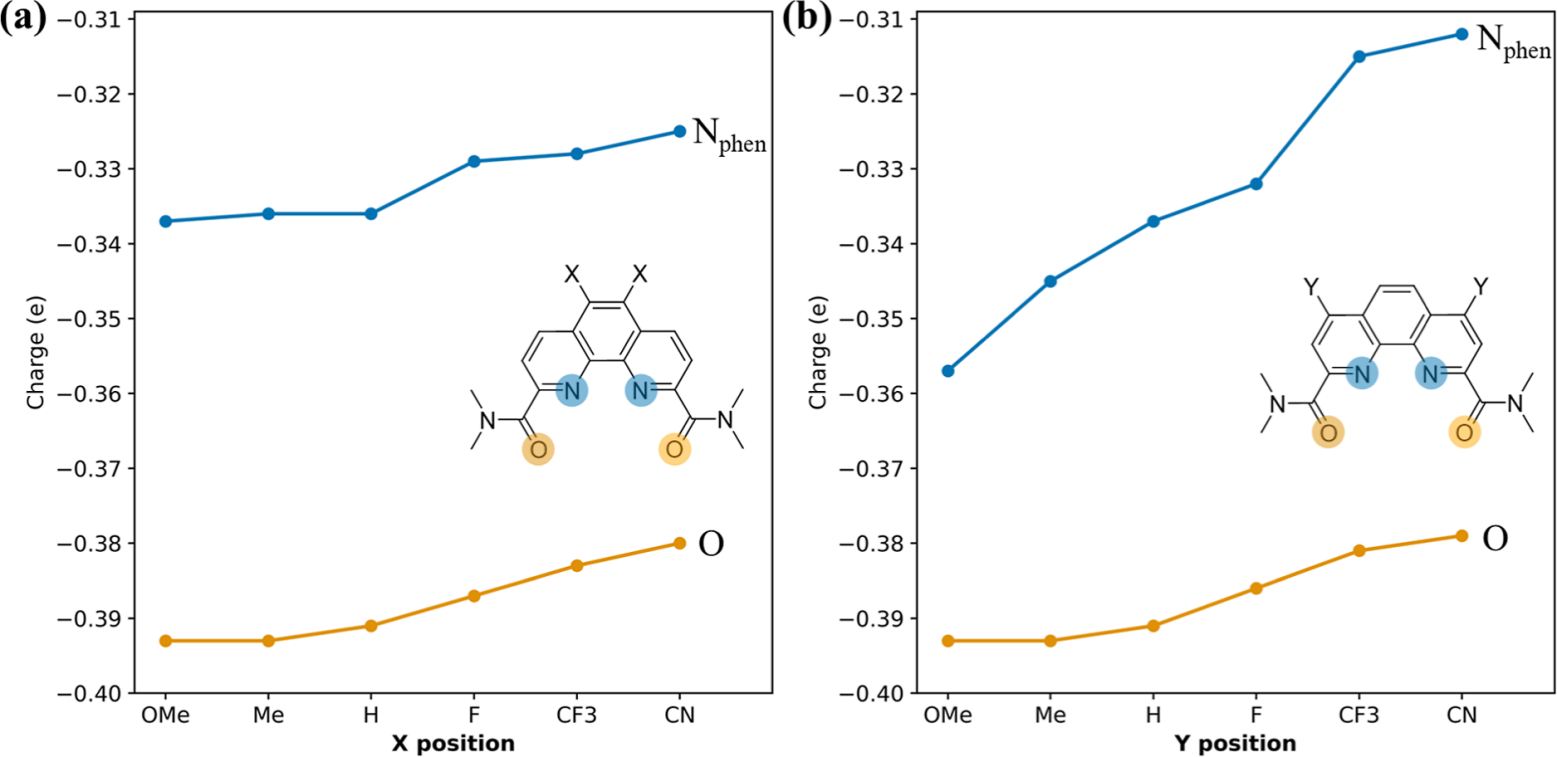
Partial atomic charges on N_phen_ and O donors of the TMeDAPhen ligands with various EDGs to EWGs: (a) at the *X* positions; (b) at the *Y* positions. Numerical values are listed in Table S1.

### 3.2 Substitution at *X* or *Y* Positions and Effects on Selectivity and Binding Energy

Next, we investigated how substituents at positions *X* and *Y* affect the binding free energy according to eq 1. The computed binding free energies across the Ln series are shown in Figure 3. One can see that for each specific ligand, the binding strength decreases from La to Lu, which can be attributed to the relatively large coordination cavity and rigidity of the ligands, which is suited well for the larger Ln ions. As the series progresses to the smaller Lu ion, the ligand must adjust its geometry, which leads to a reduction in binding strength.^60^ From ligand to ligand due to substitution at either *X* (Figure 3a) or *Y* (Figure 3b), the trendlines within each panel show that EDGs enhance the binding strength, whereas EWGs diminish it. Comparing *X* and *Y* side-by-side, we can see that substitution at the *Y* position yields a more pronounced effect on binding energy than that at the *X* position, consistent with the trends of partial atomic charges on the ligands in Figure 2.

**Figure 3:**
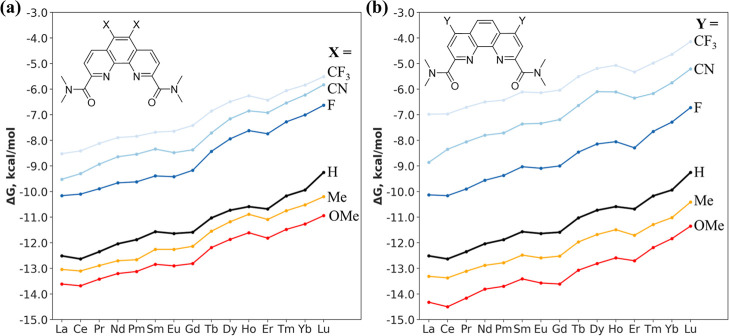
Binding energy (Δ*G*_b_, kcal/mol; eq 1) of Ln complexes with ligands possessing various EDGs or EWGs at the (a) *X* and (b) *Y* positions, computed at the PBE0-D3 level. Numerical values are listed in Tables S2 and S3.

Another way to evaluate the performance of ligands is to calculate the selectivity relative to the DAPhen reference ligand using eq 2. The resulting ΔΔ*G* values are plotted in Figure 4. For position *X* (Figure 4a), stronger EDGs (e.g., −OMe) decrease selectivity (the ΔΔ*G* range narrows), whereas stronger EWGs (e.g., −CN) increase selectivity (the ΔΔ*G* range widens). Substitution at the position *Y* (Figure 4b) follows a similar trend. Overall, the highest selectivity is observed for the −CN groups at either the *X* or *Y* position, followed by the −F group.

**Figure 4:**
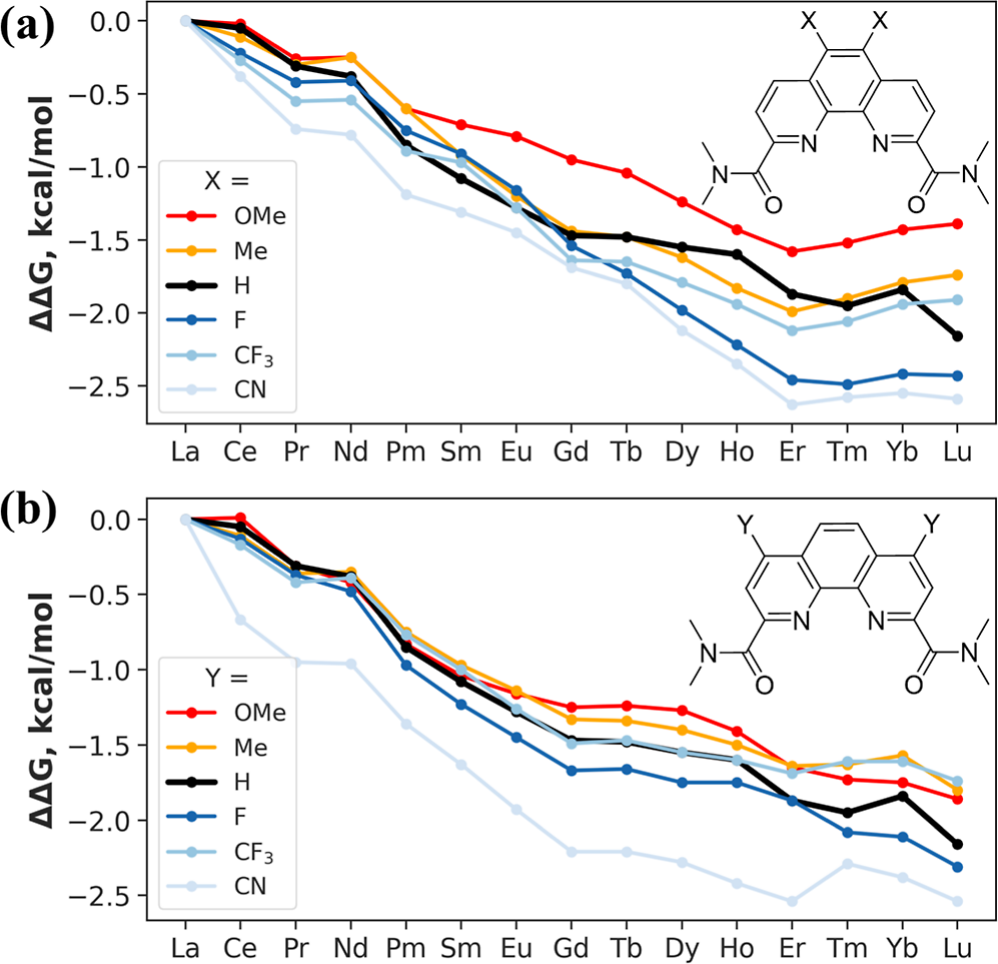
Relative aqueous-phase selectivity (ΔΔ*G*, kcal/mol; eq 2) of Ln complexes formed with ligands possessing various EDG and EWG substituents introduced at the (a) *X* and (b) *Y* positions of the ligand, computed at the PBE0-D3 level. Numerical values are listed in Tables S4 and S5.

### 3.3 QTAIM Analysis

We found that the EDGs/EWGs at the *X* and *Y* positions have a negligible influence on the geometry of Ln complexes: all bond-length variations within the first coordination sphere are within a few hundredths of an angstrom (∼0.01–0.04 Å). To better understand the electronic effects instead, we applied QTAIM bonding analysis, focusing on the three types of bonds: Ln–O­(CO), Ln–N­(Phen), and Ln–O­(nitrate), as shown in Figure 5a. The electron densities (ρ_el_) at the critical points along the three bonds for La complexes with various EDGs/EWGs at the *Y* position are plotted in Figure 5b. Higher electron density values at bond critical points (BCPs) generally correlate with stronger chemical bonds. One can see that the La–N­(Phen) bond is most sensitive to the substitution group: EDGs increase electron density and strengthen the bond, whereas EWGs decrease it. The Ln–O­(nitrate) and Ln–O­(CO) bonds are much less sensitive to the substitution groups. The QTAIM results are fully consistent with the partial atomic charges in Figure 2b that show the strongest dependence on the *Y* substituent for the N_Phen_ donor.

**Figure 5:**
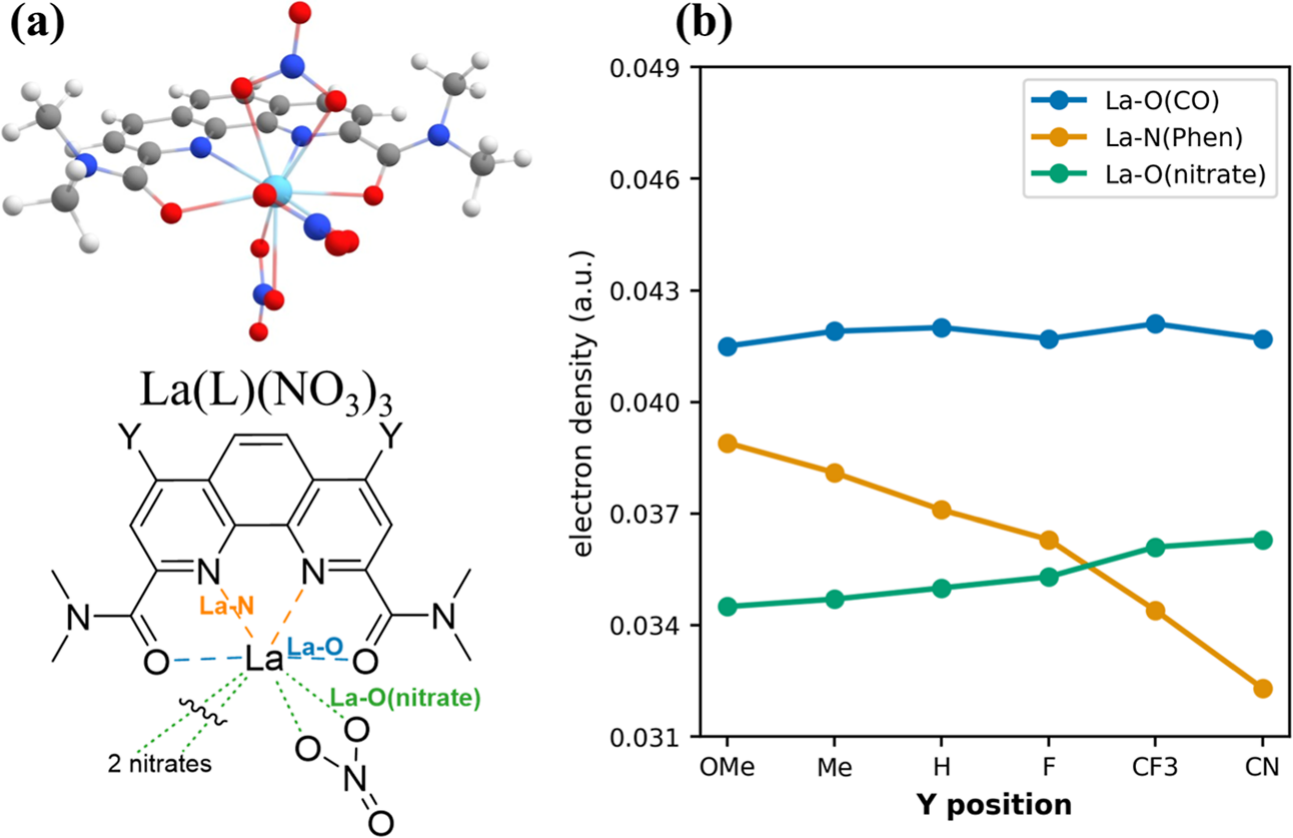
Effect of EDGs and EWGs on strength of bonds in the first coordination sphere: (a) schematic representation of three La bond types (La–N­(Phen), La–O­(CO), La–O­(nitrate)); (b) electron densities at the (3,–1) critical points of La–N­(Phen), La–O­(CO), La–O­(nitrate) bonds in La­(L)­(NO_3_)_3_ complexes with different substituent groups at the *Y* position on L. Numerical values are listed in Table S6.

We further analyzed the Laplacian of the electron density (∇^2^ρ) at the BCPs in both La and Lu complexes (see Figure S1 in the Supporting Information). This QTAIM descriptor, widely used as a robust and basis-set-independent measure of covalency, is particularly well-suited for f elements, where traditional approaches such as Mulliken and Löwdin overlap populations are notoriously basis-set-dependent and give unstable, often misleading, covalency estimates for f-block systems. Figure S1 shows that the Laplacian at the BCP of Ln-N­(Phen) becomes less positive from OMe to CN, indicating a decreasing strength of ionic interaction. While all ∇^2^ρ values remain positive, consistent with predominantly ionic closed-shell interactions, the downward trend for Ln-N­(Phen) indicates a slight increase in the covalent character, with ligands possessing stronger EWGs.

### 3.4 Substitution at Multiple Positions

We further expanded the effects of EDGs and EWGs by substituting two, four, and all six hydrogen atoms in the Phen skeleton, as shown in Figure 6a. –Me and –F groups were chosen to represent EDGs and EWGs, respectively. One can see that additional EDGs (tetra-Me) further enhance binding, while additional EWGs (hexa-F) lead to weaker binding across the Ln series (Figure 6b). More interestingly, the range of ΔΔ*G* increases to 4.0 kcal/mol for the hexa-F substitutions, indicating that the hexa-F ligand offers the best selectivity (Figure 6c). A broader ΔΔ*G* range is desirable as it corresponds to greater energetic discrimination between different lanthanides, thus facilitating more efficient separation processes. The ΔΔ*G* range of 4.0 kcal/mol for the hexa-F ligand (corresponding to a La­(III)/Lu­(III) selectivity of ∼860) is in the middle of theoretical selectivities for the mixed N, O-donor ligands, which range from 2 to 8 kcal/mol.^31,56^ In other words, although the binding strengths of Ln­(III)­s with hexa-F are weakened, the degrees of weakening vary more among the Ln­(III)­s, yielding higher selectivity. It is opposite for the tetra-Me ligand: the degrees of increase vary less among the Ln­(III)­s, yielding a lower selectivity.

**Figure 6:**
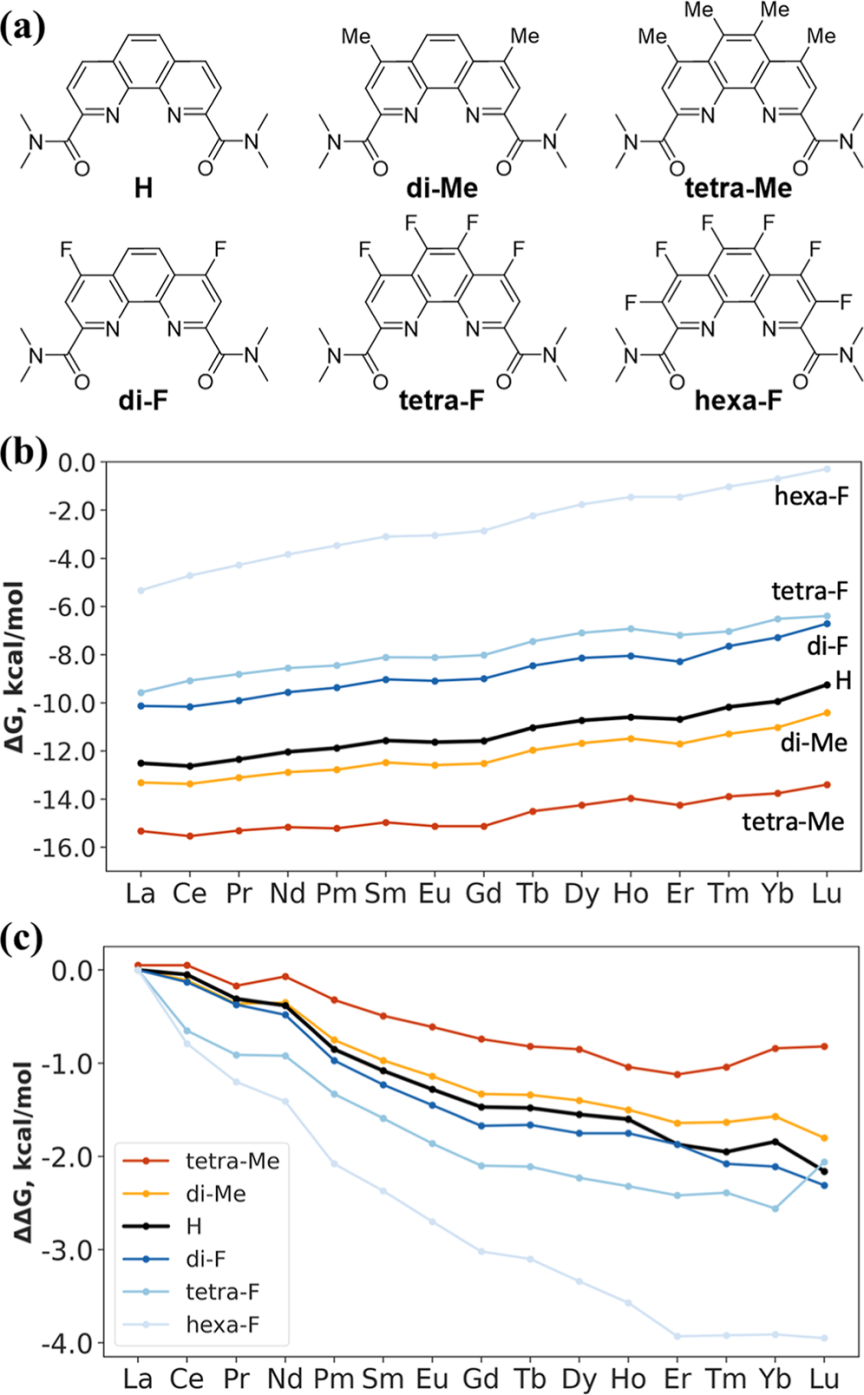
(a) Ligands with multiple Me and F substituents in the Phen ring; (b) binding energy (Δ*G*_b_, kcal/mol) of Ln complex formation; and (c) corresponding relative selectivity (ΔΔ*G*, kcal/mol). Numerical values are listed in Tables S8 and S9.

### 3.5 Implications

Experimentally, the coordination number for the 1:1 DAPhen complexes typically decreases from 10 to 9 around Ho toward the heavier lanthanides.^18,60,69^ However, standard DFT calculations using an implicit solvation model as in previous studies^39,60^ and the present work are unable to reliably capture this change in coordination number due to lanthanide contraction.^62^ Hence, as in prior computational studies aiming to reveal relative trends in binding energy and selectivity when comparing across the series, all structures were optimized with the same coordination structure in the present work (1:1 ligand to metal ratio; three bidentate nitrates; coordination number 10), which indeed shows consistent Ln-ligand bond-distance contractions (Figures S2 and S3). To address the issue of coordination number change (usually via change of nitrate/water coordination,^70^ e.g., from a bidentate nitrate to a monodentate one), future work of including explicit solvation models using ab initio molecular dynamics^71^ or polarizable force fields^72^ is needed and being pursued.

Another limitation of the present study is that the selectivity trend for Ln–ligand complexation is examined only in the aqueous phase. A complete model of lanthanide separation by solvent extraction must account for both aqueous- and organic-phase equilibria, which lies beyond the computational approach employed here. Future work should therefore focus on understanding and predicting selectivities in lanthanide separations by incorporating phase transfer and equilibrium processes between the aqueous and organic phases.

## 4 Conclusions

We investigated the influence of EDGs and EWGs substituted onto the phenanthroline backbone of DAPhen ligands on their binding energies and selectivity toward Ln­(III) ions. Through detailed computational analyses employing DFT and QTAIM, we revealed that substitution at the *Y*-position leads to a larger variation in the partial charges at the N donors (Δ*q* = 0.055 e) than at the O donors (Δ*q* = 0.015 e), while substitution at the *X*-position causes only slight variation in atomic charges at both N and O donors (Δ*q* = 0.015 e). These electronic effects alter binding strengths distinctly across the lanthanide series with EDGs generally enhancing binding energies and EWGs diminishing them. Importantly, substituents at the *Y* position exhibit a more pronounced impact compared to those at the *X* position. Our results indicate that hexafluorinated ligands exhibit superior selectivity among lanthanides, despite a decrease in absolute binding strength, highlighting the intricate relationship between electronic structure modification and lanthanide complexation selectivity. By tuning electronic properties through strategic substitution with EDGs and EWGs, we can finely control the binding affinity and selectivity profiles for lanthanides. Although substitutions predominantly affect binding energies rather than selectivity across the series, carefully selected EWGs, such as –CN and –F, demonstrate potential in enhancing selectivity, especially when substituted at multiple positions, offering a rational strategy for developing optimized ligand systems crucial for efficient lanthanide separation processes with significant technological and medical implications. Future research should focus on exploring more diverse ligand design to further enhance selectivity and binding efficiency and integrating accurate free energy simulations under realistic solvent extraction conditions to improve predictive capabilities for guiding practical separation processes.

## Supplementary Material



## Notes

Laplacian of the electron density (∇^2^ρ) at the BCPs in both La and Lu complexes; bond distance trends; and numerical values plotted in the main text (PDF)
