# Supplementary material for: Tuning Lanthanide Binding with Phenanthroline-Based Diamides via Electron-Donating and Electron-Withdrawing Groups
Source: J Phys Chem A. 2025 Oct 20;129(43):9863–70. doi: 10.1021/acs.jpca.5c03506 (PMC12581133; doi:10.1021/acs.jpca.5c03506)
Supplement: Supplementary file 1 [file jp5c03506_si_001.pdf]

Supporting Information

Tuning Lanthanide Binding with Phenanthroline-Based Diamides via Electron-Donating and Electron-Withdrawing Groups

Anton S. Pozdeev,[1] Alexander S. Ivanov,[2] Santa Jansone-Popova,[2] and De-en Jiang[1,*]

[1]Department of Chemical and Biomolecular Engineering, Vanderbilt University, Nashville, Tennessee 37235, United States

[2]Chemical Sciences Division, Oak Ridge National Laboratory, Oak Ridge, Tennessee 37831, United States

*To whom correspondence should be addressed. E-mail: de-en.jiang@vanderbilt.edu

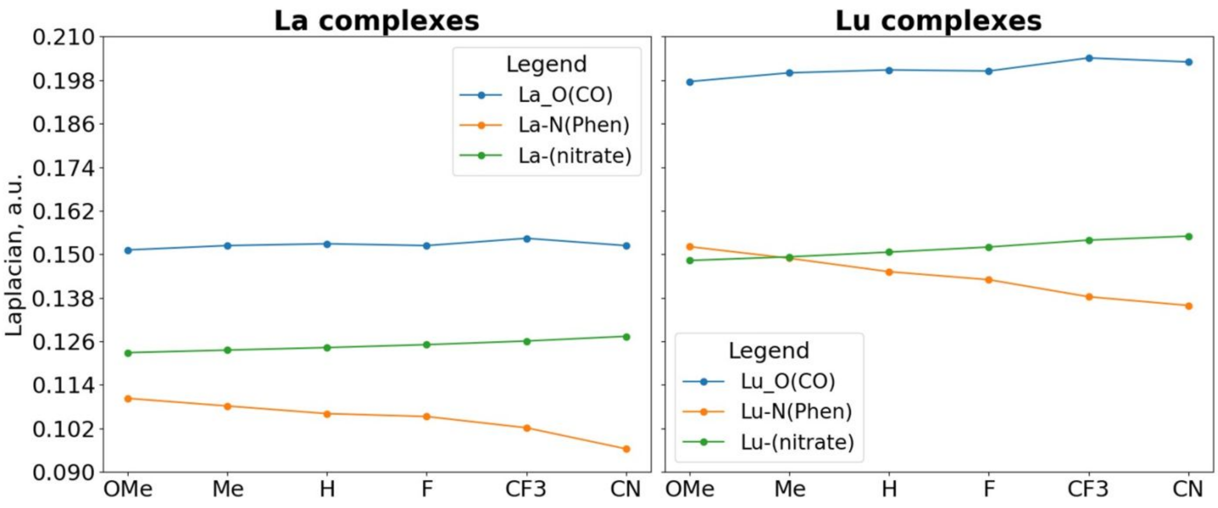

Figure S1. $\nabla^2\rho$ at the (3,–1) critical points of La–N(Phen), La–O(CO), La–O(nitrate) bonds in $La(L)(NO_3)_3$ and $Lu(L)(NO_3)_3$ complexes with different substituent groups at the Y position on the ligand L. Numerical values are listed in Table S7.

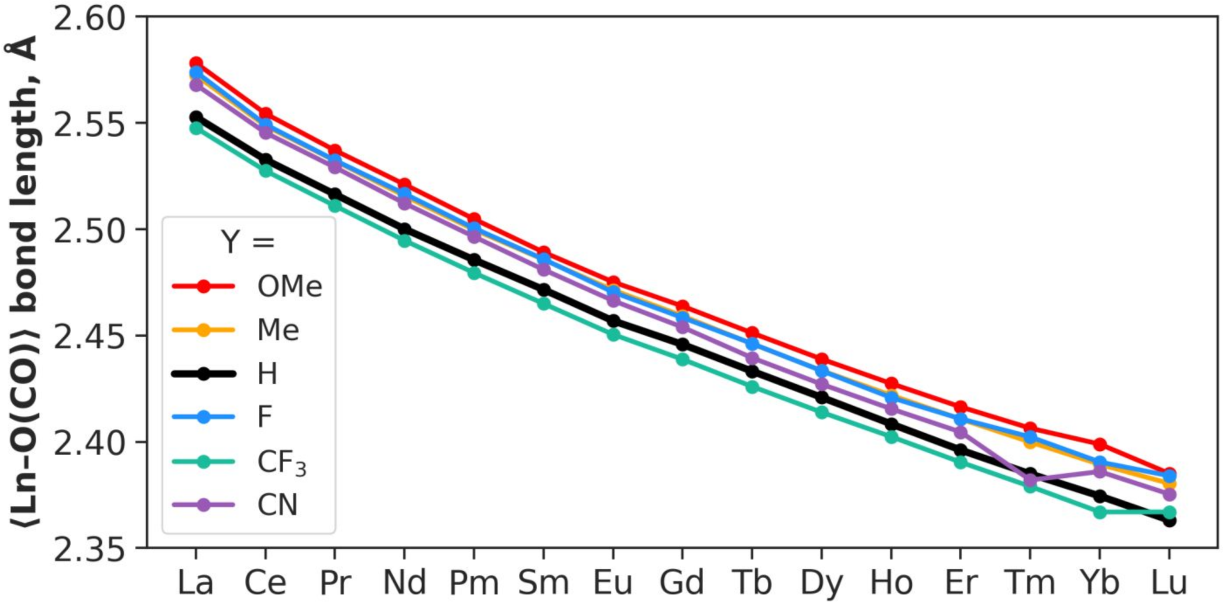

Figure S2. Variation of the average Ln–O(CO) bond lengths across the lanthanide series in Ln complexes with different substituents at the Y position.

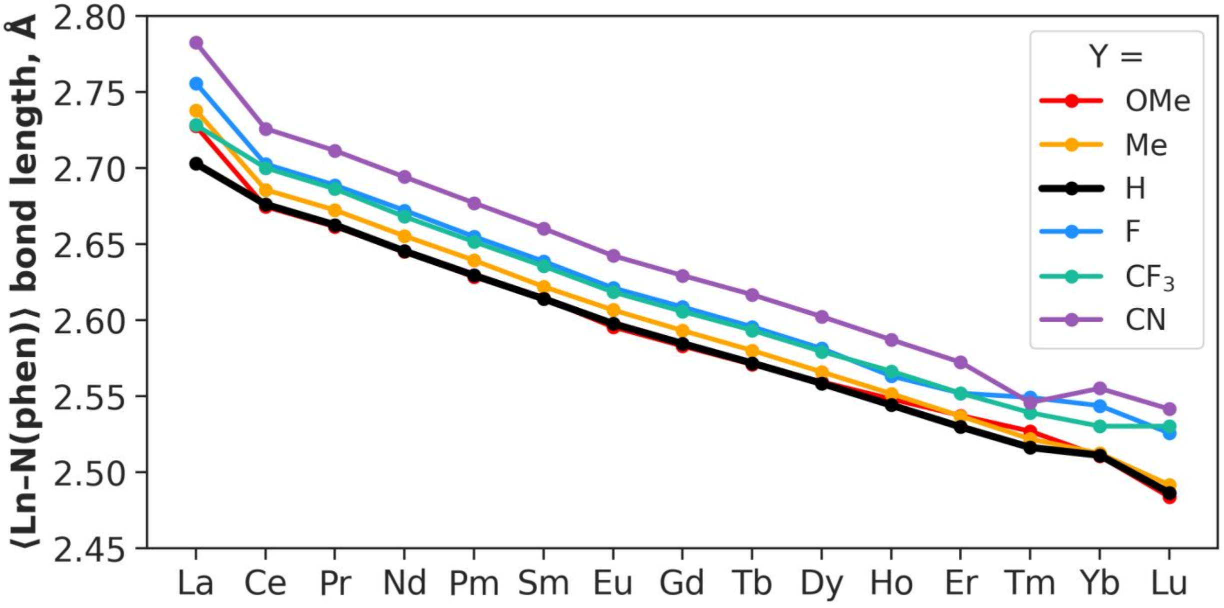

Figure S3. Variation of the average Ln–N(Phen) bond lengths across the lanthanide series in Ln complexes with different substituents at the Y position.

Table S1. Partial atomic charges on N_{phen} and O donors of the TMeDAPhen ligands with various EDGs to EWGs: at the Y/X positions.

	-OMe	-Me	-H	-F	-CF_3	-CN
N_{Phen}	-0.357/-0.337	-0.345/-0.336	-0.337/-0.337	-0.332/-0.329	-0.315/-0.328	-0.312/-0.325
O	-0.393/-0.395	-0.393/-0.395	-0.391/-0.391	-0.386/-0.387	-0.381/-0.383	-0.379/-0.380

Table S2. Binding energies (ΔG, kcal/mol), X group.

	OMe	Me	H	F	CN	CF_3
La	-13.61	-13.04	-12.51	-10.16	-9.52	-8.52
Ce	-13.68	-13.10	-12.63	-10.10	-9.30	-8.41
Pr	-13.42	-12.89	-12.35	-9.89	-8.93	-8.12
Nd	-13.20	-12.70	-12.04	-9.66	-8.64	-7.89
Pm	-13.12	-12.66	-11.88	-9.62	-8.54	-7.84
Sm	-12.84	-12.26	-11.57	-9.39	-8.34	-7.68
Eu	-12.90	-12.26	-11.64	-9.42	-8.48	-7.64
Gd	-12.81	-12.14	-11.59	-9.17	-8.37	-7.42
Tb	-12.19	-11.55	-11.03	-8.43	-7.71	-6.86
Dy	-11.87	-11.18	-10.73	-7.94	-7.16	-6.49
Ho	-11.61	-10.89	-10.59	-7.62	-6.85	-6.26
Er	-11.82	-11.09	-10.68	-7.74	-6.92	-6.43
Tm	-11.48	-10.75	-10.17	-7.28	-6.54	-6.06
Yb	-11.27	-10.52	-9.94	-7.01	-6.23	-5.84
Lu	-10.94	-10.20	-9.25	-6.63	-5.83	-5.51

Table S3. Binding energies (ΔG, kcal/mol), Y substituents.

Ln	OMe	Me	H	F	CN	CF_3
La	-14.32	-13.31	-12.51	-10.13	-8.86	-6.98
Ce	-14.50	-13.37	-12.63	-10.16	-8.35	-6.97
Pr	-14.16	-13.11	-12.35	-9.90	-8.06	-6.71
Nd	-13.81	-12.88	-12.04	-9.56	-7.80	-6.50
Pm	-13.70	-12.78	-11.88	-9.37	-7.71	-6.43
Sm	-13.41	-12.48	-11.57	-9.03	-7.36	-6.11
Eu	-13.57	-12.59	-11.64	-9.09	-7.34	-6.40
Gd	-13.61	-12.52	-11.59	-9.00	-7.19	-6.04
Tb	-13.07	-11.97	-11.03	-8.46	-6.64	-5.51
Dy	-12.81	-11.68	-10.73	-8.14	-6.10	-5.19
Ho	-12.59	-11.49	-10.59	-8.05	-6.11	-5.07
Er	-12.70	-11.71	-10.68	-8.29	-6.35	-5.33
Tm	-12.19	-11.29	-10.17	-7.65	-6.17	-4.98
Yb	-11.84	-11.02	-9.94	-7.29	-5.75	-4.64
Lu	-11.35	-10.41	-9.25	-6.72	-5.21	-4.14

Table S4. Relative selectivity values (ΔΔG, kcal/mol), Y substituents.

	OMe	Me	H	F	CN	CF_3
**La**	0.00	0.00	0.00	0.00	0.00	0.00
**Ce**	0.01	-0.11	-0.05	-0.13	-0.67	-0.17
**Pr**	-0.31	-0.36	-0.31	-0.37	-0.95	-0.42
**Nd**	-0.42	-0.35	-0.38	-0.48	-0.96	-0.39
**Pm**	-0.83	-0.75	-0.85	-0.97	-1.36	-0.77
**Sm**	-1.04	-0.97	-1.08	-1.23	-1.63	-1.00
**Eu**	-1.16	-1.14	-1.28	-1.45	-1.93	-1.26
**Gd**	-1.25	-1.33	-1.47	-1.67	-2.21	-1.49
**Tb**	-1.24	-1.34	-1.48	-1.66	-2.21	-1.47
**Dy**	-1.27	-1.40	-1.55	-1.75	-2.28	-1.55
**Ho**	-1.41	-1.50	-1.60	-1.75	-2.42	-1.60
**Er**	-1.65	-1.64	-1.87	-1.87	-2.54	-1.69
**Tm**	-1.73	-1.63	-1.95	-2.08	-2.29	-1.61
**Yb**	-1.75	-1.57	-1.84	-2.11	-2.38	-1.61
**Lu**	-1.86	-1.80	-2.16	-2.31	-2.54	-1.74

Table S5. Relative selectivity values (ΔΔG, kcal/mol), X substituents.

	OMe	Me	H	F	CN	CF_3
**La**	0.00	0.00	0.00	0.00	0.00	0.00
**Ce**	-0.02	-0.11	-0.05	-0.22	-0.38	-0.27
**Pr**	-0.26	-0.30	-0.31	-0.42	-0.74	-0.55
**Nd**	-0.25	-0.25	-0.38	-0.41	-0.78	-0.54
**Pm**	-0.60	-0.60	-0.85	-0.75	-1.19	-0.89
**Sm**	-0.71	-0.92	-1.08	-0.91	-1.31	-0.97
**Eu**	-0.79	-1.20	-1.28	-1.16	-1.45	-1.28
**Gd**	-0.95	-1.44	-1.47	-1.54	-1.69	-1.64
**Tb**	-1.04	-1.48	-1.48	-1.73	-1.80	-1.65
**Dy**	-1.24	-1.62	-1.55	-1.98	-2.12	-1.79
**Ho**	-1.43	-1.83	-1.60	-2.22	-2.35	-1.94
**Er**	-1.58	-1.99	-1.87	-2.46	-2.63	-2.12
**Tm**	-1.52	-1.90	-1.95	-2.49	-2.58	-2.06
**Yb**	-1.43	-1.79	-1.84	-2.42	-2.55	-1.94
**Lu**	-1.39	-1.74	-2.16	-2.43	-2.59	-1.91

Table S6. Average electron density (ρ, a.u.) at bond (3, -1) critical points for La and Lu complexes, Y-substituents.

Y=	La complexes			Lu complexes		
	N_{Phen}	O_{CO}	$O_{nitrate}$	N_{Phen}	O_{CO}	$O_{nitrate}$
**OMe**	0.0389	0.0415	0.0345	0.0459	0.0468	0.0366
**Me**	0.0381	0.0419	0.0347	0.0449	0.0474	0.0369
**H**	0.0371	0.0420	0.0350	0.0437	0.0476	0.0373
**F**	0.0363	0.0417	0.0353	0.0427	0.0474	0.0377
**CF_3**	0.0348	0.0424	0.0357	0.0409	0.0483	0.0383
**CN**	0.0323	0.0417	0.0363	0.0401	0.0479	0.0386

Table S7. Average Laplacian of electron density ($\nabla^2\rho$, a.u.) at bond (3, -1) critical points for La and Lu complexes, Y-substituents.

Y =	La complexes			Lu complexes		
	N_{Phen}	O_{CO}	$O_{nitrate}$	N_{Phen}	O_{CO}	$O_{nitrate}$
**OMe**	0.1103	0.1512	0.1229	0.1521	0.1976	0.1483
**Me**	0.1082	0.1524	0.1236	0.1489	0.2000	0.1493
**H**	0.1061	0.1529	0.1243	0.1452	0.2008	0.1506
**F**	0.1053	0.1524	0.1251	0.1430	0.2005	0.1520
**CF3**	0.1022	0.1544	0.1261	0.1383	0.2041	0.1539
**CN**	0.0964	0.1524	0.1274	0.1359	0.2030	0.1550

Table S8. Binding energies (ΔΔG, kcal/mol), multiple Me and F substituents.

	tetra-Me	di-Me	H	di-F	tetra-F	hexa-F
**La**	-15.33	-13.31	-12.51	-10.13	-9.57	-5.34
**Ce**	-15.54	-13.37	-12.63	-10.16	-9.08	-4.72
**Pr**	-15.31	-13.11	-12.35	-9.90	-8.81	-4.28
**Nd**	-15.17	-12.88	-12.04	-9.56	-8.56	-3.84
**Pm**	-15.22	-12.78	-11.88	-9.37	-8.45	-3.47
**Sm**	-14.97	-12.48	-11.57	-9.03	-8.11	-3.10
**Eu**	-15.13	-12.59	-11.64	-9.09	-8.12	-3.05
**Gd**	-15.13	-12.52	-11.59	-9.00	-8.02	-2.86
**Tb**	-14.50	-11.97	-11.03	-8.46	-7.45	-2.23
**Dy**	-14.25	-11.68	-10.73	-8.14	-7.10	-1.76
**Ho**	-13.97	-11.49	-10.59	-8.05	-6.93	-1.45
**Er**	-14.25	-11.71	-10.68	-8.29	-7.19	-1.45
**Tm**	-13.89	-11.29	-10.17	-7.65	-7.04	-1.02
**Yb**	-13.76	-11.02	-9.94	-7.29	-6.52	-0.70
**Lu**	-14.30	-10.41	-9.25	-6.72	-6.40	-0.29

Table S9. Relative selectivity values (ΔΔG, kcal/mol), multiple Me and F substituents.

	**tetra-Me**	**di-Me**	**H**	**di-F**	**tetra-F**	**hexa-F**
**La**	0.00	0.00	0.00	0.00	0.00	0.00
**Ce**	0.05	-0.11	-0.05	-0.13	-0.65	-0.79
**Pr**	-0.17	-0.36	-0.31	-0.37	-0.91	-1.20
**Nd**	-0.07	-0.35	-0.38	-0.48	-0.92	-1.41
**Pm**	-0.32	-0.75	-0.85	-0.97	-1.33	-2.08
**Sm**	-0.49	-0.97	-1.08	-1.23	-1.59	-2.37
**Eu**	-0.61	-1.14	-1.28	-1.45	-1.86	-2.70
**Gd**	-0.74	-1.33	-1.47	-1.67	-2.10	-3.02
**Tb**	-0.82	-1.34	-1.48	-1.66	-2.11	-3.10
**Dy**	-0.85	-1.40	-1.55	-1.75	-2.23	-3.34
**Ho**	-1.04	-1.50	-1.60	-1.75	-2.32	-3.57
**Er**	-1.12	-1.64	-1.87	-1.87	-2.42	-3.93
**Tm**	-1.04	-1.63	-1.95	-2.08	-2.39	-3.92
**Yb**	-0.84	-1.57	-1.84	-2.11	-2.56	-3.91
**Lu**	-0.82	-1.80	-2.16	-2.31	-2.06	-3.95
